# Atypical Granulomatosis with Polyangiitis Presenting with Meibomitis, Scleritis, Uveitis and Papillary Bladder Tumor: A Case Report and Literature Review

**DOI:** 10.3390/diagnostics11040680

**Published:** 2021-04-09

**Authors:** Takashi Kojima, Murat Dogru, Eisuke Shimizu, Hiroyuki Yazu, Aya Takahashi, Jun Shimazaki

**Affiliations:** 1 Department of Ophthalmology, Keio University School of Medicine, Tokyo 160-8582, Japan; kojima_takashi@keio.jp (T.K.); ophthalmolog1st.acek39@gmail.com (E.S.); g.h.yazu@gmail.com (H.Y.); 2 Department of Ophthalmology, Tokyo Dental College Ichikawa General Hospital, Chiba 272-8513, Japan; ayatak1126@gmail.com (A.T.); jun@eyebank.or.jp (J.S.); 3 Department of Ophthalmology, Tsurumi University School of Dental Medicine, Kanagawa 230-8501, Japan

**Keywords:** granulomatosis with polyangiitis, meibomian gland dysfunction, mibomitis, papillary bladder tumor, Wegener’s granulomatosis

## Abstract

Granulomatosis with polyangiitis (GPA) presents with a variety of systemic findings, sometimes with ocular findings initially, but is often difficult to diagnose at an early stage. An 85-year-old male had complaints of ocular dryness and redness and was diagnosed with meibomian gland dysfunction with meibomitis. Despite an initial treatment with topical steroid and antibiotics, the meibomitis did not improve and the left eye developed scleritis and iridocyclitis. The patient was administered topical mydriatics and oral steroids. During follow-up, the patient developed left hearing difficulty and reported a darker urine. Urinalysis revealed microscopic hematuria. A blood test showed an elevated erythrocyte sedimentation rate, positivity for perinuclear anti-neutorophil cytoplasmic antibody, and elevations in blood urea nitrogen and serum creatinine. Nasal mucosal biopsy showed a non-necrotizing granulomatous inflammation. Renal biopsy revealed focal glomerulosclerosis. Cystoscopy and bladder wash followed by a planned transurethral resection revealed atypical cells and apical papillary tumors which were resected. Iridocyclitis and scleritis responded well to oral prednisolone with 0.1% topical betamethasone and prednisolone ointment. The patient is tumor free with no recurrences 24 months after resection. GPA may present atypically with meibomian gland dysfunction without showing representative clinical findings. Early detection and treatment are essential for visual recovery.

## 1. Introduction

Granulomatosis with polyangiitis (GPA: also known as Wegener’s granulomatosis) presents with diverse manifestations and the early diagnosis may be difficult in cases with atypical findings. Reported classical ocular findings of GPA include proptosis, orbital pseudotumor, and nasolacrimal duct obstruction. Patients may develop focal vasculitis in either the anterior or posterior segment of the eye resulting in chalazion, conjunctivitis, episcleritis, scleritis, peripheral ulcerative keratitis, uveitis and, much more rarely, optic nerve vasculitis or retinal artery occlusions [1]. Upper airway disease appears to be the most common initial feature in this entity. Ocular signs may be the initial finding in approximately 16% of the cases. It is much rare to observe peripheral ulcerative keratitis or necrotizing scleritis as the initial presentation [1]. The presence of these conditions is important since they indicate the inflammatory involvement of small vessels.

Meibomian gland dysfunction (MGD) is one of the risk factors for developing dry eye [2,3,4]. According to the international MGD workshop report [5], MGD is divided into hyposecretory, obstructive and hypersecretory types with each one of them being classified into primary and secondary types. Among subtypes of MGD, it is reported that the obstructive type that occurs in elderly patients is a major subtype of MGD [5,6,7]. Meibomitis and posterior blepharitis with lid margin telangiectasia may be accompanied with MGD. However, it is not clear how meibomian gland inflammation is involved in the pathology. Various factors such as topical medications, dietary intake, ocular surface microbiome, demodex, contact lens use, and aging are involved in the onset of MGD [8,9,10,11]. Among systemic and congenital diseases, Turner syndrome, diabetes and Sjogren syndrome have been reported to be associated with the onset of MGD [8,12,13].

We experienced a case of GPA initially presenting only with obstructive meibomian gland disease accompanied with meibomitis. This case later developed scleritis and granulomatous unilateral uveitis, the final work up of whom also revealed a papillary bladder tumor.

## 2. Report of a Case

**Figure 1:**
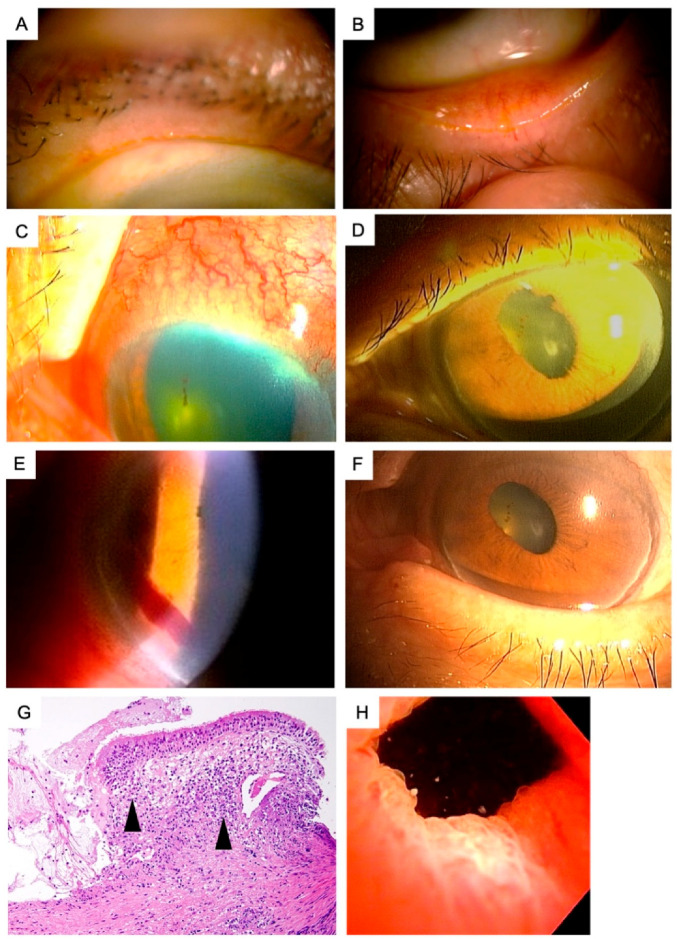
Anterior segment findings, nasal mucosal histopathology and bladder apex findings in patient with atypical Granulomatosis with polyangiitis. (**A**,**B**) slit lamp anterior segment findings at the initial visit. Note the presence of pouting and meibomian gland orifice obstruction, lid margin telangiectasia and turbid nature of meibum (OS). (**C**,**D**) The slit lamp anterior segment findings at the second week of follow up. Note the scleritis and marked conjunctival injection (**C**), the anterior chamber inflammation and anterior synechiae (**D**), and the anterior chamber bleeding presumably due to vasculitis of iris vessels (**E**). (**F**) The slit lamp anterior segment findings at the final visit. Note the absence of ciliary injection, lack of anterior chamber inflammation and meibomian gland dysfunction. Iris pigment deposits was remained on the anterior lenticular surface after relieving the anterior synechiae with mydriatics. (**G**) Nasal mucosal histopathology specimens. Note the mucosal and submucosal inflammatory cells (arrow heads) consisting mainly of neutrophils in the Hematoxylin and Eosin stainings. (**H**) The digital photograph of the bladder apex during trans urethral resection. Note the epithelial multifocal papillary tumors.

An 85-year-old Japanese male was seen due to complaints of dryness and redness of the eyes and was diagnosed with bilateral cataracts, conjunctival laxity and obstructive meibomian gland dysfunction. He had no systemic and ocular diseases and was not on any ocular or systemic medications. His initial ophthalmologic examination disclosed corrected visual acuities of 20/20 OD and 14/20 OS. His intraocular pressures were 14 mmHg OD and 12 mmHg OS. Slit lamp examination revealed bilateral cataract and bilateral upper and lower lid obstructive meibomian gland dysfunction with meibomitis (Figure 1A,B). Altered expressibility of meibum, telangiectasia at the lid margin, plugging, and pouting signs were observed. From these findings, Grade 3 MGD was diagnosed according to the Tear Film and Ocular Surface (TFOS) MGD workshop classification [7]. The tear film breakup times were 2 s OU and the fluorescein staining scores were 3 pts OD and 4 pts OS (min: 0 pts; max: 9 points). His fundoscopy showed peripapillary atrophy and a generalized retinal pigment atrophy in both eyes. He underwent upper and lower lid expression, was recommended to apply eye warmer masks for 10 min twice a day and was prescribed 0.1% topical fluorometholone q.i.d and 0.3% ofloxacin q.i.d for 2 weeks. At the control visit 2 weeks later, the meibomitis had slightly improved but the left eye was observed to develop scleritis (Figure 1C), severe iridocyclitis with mutton fat keratic precipitates, ++ cells in the anterior chamber as well as anterior synechiae (Figure 1D) and slight anterior chamber bleeding without a history of ocular rubbing or trauma (Figure 1E). The left corrected visual acuity was 6/20. The patient was administered 1% topical tropicamide t.i.d and 0.1% topical betamethasone q.i.d. and was re-examined three days later. An increase in anterior chamber cells and the appearance of a plasma exudate prompted use of 20 mg/day of oral prednisolone. The patient was then referred to the Department of Ophthalmology, Tokyo Dental College Ichikawa General Hospital for further investigation. A full blown diagnostic work-up for uveitis were carried out including complete blood count, urinary analyses, liver enzymes, renal function tests including creatinine and blood urea nitrogen (BUN), chest roentgen, electrocardiogram, thyroid gland markers including free T3, T4, thyroid stimulating hormone (TSH), anti-TSH antibody, anti-thyroid peroxidase (TPO) antibody, markers for connective tissue diseases such as angiotensin converting enzyme (ACE), anti-nuclear antibody (ANA), anti-single strand DNA antibody, rheumatoid factor, anti-cyclic citrullinated peptide (CCP) antibody, cytoplasmic anti-neutrophil cytoplasmic antibody (C-ANCA), perinuclear-ANCA (P-ANCA), infection markers including serology for tuberculosis, syphilis, toxoplasma, toxocara, hepatitis B/C, herpes simplex/zoster and HIV. A laboratory work up for uveitis revealed elevated BUN (21.4 mg/dL), serum creatinine (1.28 mg/dL), Perinuclear anti-neutorophil cytoplasmic antibody (P-ANCA) levels (5.3 U/mL) and prostate specific antigen (PSA) level (PSA > 4 ng/mL). The C-ANCA level was within normal limits (1.4 U/mL [normal <2.0 U/mL]). The serum ACE was also normal (9.6 IU/L). The interferon-gamma release assays (T-SPOT) test was negative. His ECG did not reveal any arrythmias. A chest X-ray did not show any infiltration, mass lesion or bilateral hilar lymphadenopathy. Thoracic CT scan showed right main bronchial wall thickening but no infiltrations or mass lesions. The urinalysis showed microscopic hematuria (<100 rbc/HPF) which prompted a consultation with Nephrology and Urology Departments. The patient was scheduled for a renal biopsy upon a tentative diagnosis of polyangiitis and was hospitalized. The patient was also scheduled for cystoscopy upon the urine cytology work up showing atypical cells. Administration of oral steroids resulted in clearance of cells from the anterior chamber, the plasma exudate, regression of ciliary injection and cessation of a further progression in anterior synechiae. During hospitalization, the patient reported hearing difficulty on the left side. ENT department consultation revealed left sensorineural hearing loss. A nasal biopsy showed non-granulamatous inflammatory infiltrates (Figure 1G). A broncoscopy aided bronchial biopsy and lavage disclosed inflammatory infiltrates mainly consisting of neutrophils and no evidence of malignancy or atypical cells. The ENT department advised an increase of oral prednisolone to 30 mg/day for the hearing loss. This dosage was effective but resulted in generalized muscular weakness and walking difficulty in the patient. The renal biopsy did not show necrotizing granulomatous angiitis but revealed areas of focal glomerulosclerosis. Cystoscopy showed epithelial tumor with papillary formations at the bladder apex (Figure 1H) for which a transurethral resection (TUR) of the tumor and intravesicular bacillus Calmette-Guerin (BCG) wash was performed. An interdisciplinary consultation on the overall findings led to a final diagnosis of atypical GPA. While all physicians following the case believed that 1 mg/kg/day of oral prednisolone and oral cyclophosphamide would have been better for the general prognosis, the improvement of hearing loss and renal functions with 30 mg/day, the presence of a malignancy and general muscular weakness, it was agreed that a further increase in steroid dose or addition of oral chemotherapeutics would not be necessary. Upon improvement of the hearing loss, the oral corticosteroids were tapered off and discontinued over a period of five months after the first prescription (30 mg/day for 1 month, 25 mg/day for 2 month, 20 mg/day for 1 month, 10 mg/day for 1 month). Three years into the follow up since the diagnosis, the patient is free of any recurrences of the bladder tumor or relapses in hearing loss. His final ophthalmologic examination revealed corrected visual acuities of 16/20 OD and 8/20 OS, bilateral cataracts with progression of the left lenticular opacity. The final intraocular pressures were 12 mmHg OU. Ciliary injection had resolved with no cells in the anterior chamber with fine keratic precipitates and iris pigment deposits on the anterior surface of the lens (Figure 1F). Anterior synechia were observed between 7–9 o’clock on the lens surface. Expressibility and quality of meibum became normal, and telangiectasia at lid margin, plugging and pouting signs disappeared. After treatment with GPA, the severity of MGD improved from grade 3 to grade 0 according to the TFOS MGD classification. The patient was on 0.1% fluorometholone t.i.d, and 1% topical tropicamide b.i.d at the final visit. Since the patient was free of any uveitis relapses for the last 2 years, he was being scheduled for cataract surgery.

## 3. Discussion

Granulomatosis with polyangiitis (GPA: also known as Wegener’s granulomatosis) is an autoimmune disease with a prevalence of 3/100,000 in the general population [14]. Classical GPA presents with necrotizing granulomas of the upper and lower respiratory tracts, systemic vasculitis and necrotizing glomerulonephritis. The atypical or the limited form may start with any other organ involvement. The most recognizable and earliest patient complaints for seeking medical care are purulent nasal discharge, epistaxis, nasal ulceration, otitis media, saddle nose defect, cough, hemoptysis, dyspnea or chest pain none of which were observed in our patient [15]. The current patient presented with the initial findings of obstructive meibomian gland disease with meibomitis with dry eyes. The patient later developed scleritis and unilateral iridocylitis which prompted a uveitis work-up. Elevation of P-ANCA (10–15% of the cases; C-ANCA elevated in 80–95%) levels, perturbation of renal functions with elevations in serum, PSA and creatinine and BUN levels together with the presence of microscopic hematuria increased the suspicion for a polyangiitis for which reason the patient was referred to Nephrology and Urology Departments.

While further investigation did not show common features of presentation such as pulmonary infiltrates present in nearly 50% of the initial cases or renal lesions (which are clinically evident in only 11–20% of the patients) suggestive of necrotizing glomerulonephritis, focal glomerulosclerosis, a rare associate of GPA was noted [1,15]. Development of unilateral sensorineural deafness together with the evidence from inflammatory infiltrates in the nasal mucosa biopsy samples further increased the suspicion for an atypical presentation of GPA which could be effectively managed by 30 mg/day of oral corticosteroid therapy. It should also be noted that GPA is associated with an increased incidence of renal cell carcinoma or bladder carcinoma which may be present before the onset or may be induced by cyclophosphamide treatment. Most adverse, nonfatal outcomes are related to the treatment of GPA [14,16]. These include side effects from glucocorticoids, increased risk of malignancy, and progressive organ failure [1,15]. Patients with GPA have an increased risk of deep vein thrombosis and pulmonary embolism, probably because of the nature of the vasculitis. Progressive renal failure with kidney involvement and respiratory failure with pulmonary involvement can occur. Untreated patients have a low survival rate of only 20% at 2 years. However, the 2-year survival rate for treated patients is about 90% [1,15]. Timely diagnosis and resection of the intraepithelial tumor before local or systemic metastasis definitely had a favorable impact on the survival and the clinical outcome of the patient. The decision not to include cyclophosphamide in the protocol and tailoring the steroid dose according to the clinical course of this patient might have also helped with the overall favorable general outcome.

Ocular or orbital involvement is not very common and seen in 15% of patients at presentation and in up to 50% of patients during the course of the disease [15,17]. The reported ocular findings are summarized in Table 1 [18,19,20,21,22,23,24,25,26,27,28,29,30,31,32,33,34,35,36,37,38,39,40].

To our knowledge, A PubMed search using the key words polyangiitis, Wegener’s granulomatosis, ocular complications, meibomian gland disease did not reveal any cases associated with obstructive MGD initially while chalazion or blepharitis were described as associated findings in a case report earlier [40,41]. It has been reported that the secretion of elastases and lipases by bacteria in the eyelids and meibomian glands increases fatty free acid in meibum, which increases the viscosity of meibum and obstructs meibomian gland [42,43]. Subsequently, the increase in pressure in the meibomian gland due to occlusion causes inflammation and hyperkeratinization of meibomian gland duct epithelium. In this case, the addition of systemic steroids resulted in the resolution of MGD, scleritis and uveitis gradually, which led us to hypothesize that MGD was related to inflammation and vasculitis induced by GPA. Although a lid biopsy was not allowed due to Ethic Board regulations in our case, the clinical course and improvement of ocular findings only after systemic steroid use suggest an association with GPA. In ANCA-associated vasculitis, neutrophil extracellular traps (NETs), a bactericidal mechanism of neutrophils, have been shown to promote myeloperoxidase-ANCA production, thrombosis, inflammation and fibrosis, leading to a vicious cycle of inflammatory tissue changes [44,45,46,47]. In a recent study, NETs were found in the ocular discharge of patients with blepharitis, and extensive aggregation of NETs were found in the meibomian glands [48]. In addition, in a murine model of allergic eye disease, it has been shown that NETs accumulate in the meibomian glands, obstructing the ducts and causing meibum retention and subsequent acinar atrophy [48]. Although histopathological investigation of meibomian glands could not be performed in our case, based on the results of these recent studies, we believe that aggregated NETs might have caused MGD in this case. The role of vascular inflammation in MGD needs to be elucidated by analysis of animal models and human samples in the future.

Scleritis and iridocyclitis could not be managed effectively with topical bethamethasone and mydriatics until the administration of 20 mg/day of oral prednisone which could eliminate the intraocular inflammation successfully. Oral steroids also helped in preventing further recurrences during the follow-up.

In conclusion, GPA may present atypically with dry eyes due to meibomian gland disease and or with anterior uveitis and scleritis without any of the reported representative clinical findings. A timely diagnosis and treatment of associated scleritis and uveitis with systemic steroids may be rewarding for visual recovery. Close interdisciplinary consultation is extremely important and may disclose life threatening urinary tract tumors as in the current case, the early resection of which improved the clinical outcome.

## Figures and Tables

**Table 1:** Ocular manifestations of Wegener granulomatosis.

Tissue	Diseases
Orbital Involvement (45–50%)	Dacryoadenitis, Orbital Myositis, Orbital Pseudotumor
Eyelids, Lacrimal system and the conjunctiva (16%)	Dacryoadenitis, Dacryocystitis, Nasolacrimal duct obstruction, Ptosis, Lid granuloma, Chalazion, Entropion, Trichiasis, Xanthelasma, Cicatrizing conjunctivitis, Conjunctival ulceration/necrosis
Sclera (16–38%)	Diffuse, nodular or necrotizing anterior scleritis, Posterior scleritis
Cornea (0–40%)	Peripheral ulcerative keratitis, Exposure keratopathy, Infectious keratitis, Corneal perforation
Uvea (0–10%)	Unilateral or bilateral anterior, intermediate or posterior uveitis
Retina and choroid	Unilateral or bilateral, central or multifocal vasculitis, Retinitis, Chorioretinitis, Macular edema, Exudative retinal detachment, Retinal necrosis, Retinal vein occlusion
Neuroophthalmic manifestations	Optic neuritis/perineuritis, Compressive optic neuropathy, Oculomotor, trochlear and abducens nerve palsies, Horner’s syndrome

## Data Availability

Data sharing not applicable.
